# Navigating independence: the short-term and long-term impacts of USAID dismantling on Somalia’s humanitarian and development landscape

**DOI:** 10.1136/bmjgh-2025-019330

**Published:** 2025-05-22

**Authors:** Abdirahman Moallim Ibrahim, Mohamed Osman Mohamed

**Affiliations:** 1 Jazeera University, Mogadishu, Somalia; 2 University for Peace, Ciudad Colón, San José, Costa Rica

**Keywords:** Global Health, Health policies and all other topics

**Summary box**

- Somalia’s humanitarian and development efforts heavily rely on U.S. Agency for International Development’s (USAID’s) US$1.1 billion annual support, addressing health, food security and governance, but the implications of its dismantling remain unexplored.
- This commentary highlights the immediate and long-term risks of USAID’s withdrawal, including worsened health and food crisis and identifies opportunities for Somalia to achieve self-reliance, domestic reforms and domestic diversification.
- This commentary urges Somali-led policy reforms and advocates for international support to foster local ownership, potentially reshaping aid strategies in fragile states.

As we stand on the crossover of a new era in international aid policy, the decision by the Trump administration to dismantle the U.S. Agency for International Development (USAID) sends ripples through nations like Somalia, which have long depended on USAID support for both humanitarian relief and development.^1^ As President Trump said, “We must prioritise American interests and reduce wasteful overseas spending”, this policy shift risks destabilising a nation already grappling with decades of conflict, climate shocks and political instability.^2 3^ This commentary aims to focus on the short-term and long-term implications of this policy change, emphasising the urgent imperative for Somalia to forge a path towards self-reliance, transforming a potential crisis into an opportunity for sustainable nation-building.

For years, USAID has been a cornerstone of life-saving assistance, providing critical funding and operational support across multiple vital areas. A significant cessation or reduction immediately threatens to intensify existing humanitarian crises. USAID’s contributions have been vital, with annual investments exceeding US$1.1 billion aimed at supporting food security, health services and emergency aid.^4^ The health sector, for example, has been profoundly supported by USAID, which has invested in primary healthcare services, maternal and child health programmes and disease prevention and control initiatives, reaching approximately 3–5 million women and children.^5^,^7^ Withdrawal will likely lead to gaps in service provision, potentially reversing hard-won gains in combating infectious diseases like measles, malaria, tuberculosis and HIV/AIDS and undermining fragile progress in reducing maternal and infant mortality rates. Similarly, Somalia’s precarious food security situation, perpetually threatened by drought, displacement and conflict, has been significantly addressed through USAID’s food assistance programmes.^8 9^ These programmes often represent a lifeline for millions, for instance, specific USAID funding announcements aimed to avert famine reached values of over US$400 million in critical periods.^8^ A cessation in this aid could exacerbate malnutrition rates, particularly among vulnerable populations like internally displaced persons and children, potentially pushing more Somalis into acute food insecurity and famine-like conditions.

USAID has also been instrumental in providing Water, Sanitation and Hygiene (WASH) services, crucial for preventing waterborne diseases and maintaining public health in a country where access to clean water remains below 50% for large parts of the population.^10^ The dismantling of WASH programmes could lead to increased outbreaks of preventable diseases, further straining Somalia’s already weak health infrastructure. Emergency relief efforts, responding to recurring climate-induced disasters and conflict displacement, also heavily rely on USAID funding and logistical support. The diminished capacity to respond swiftly and effectively to crises will undoubtedly increase human suffering and potentially destabilise already volatile regions.

Beyond the immediate humanitarian crisis, the long-term developmental implications of USAID’s withdrawal are equally concerning. USAID’s development assistance has extended beyond emergency relief, focusing on building longer-term resilience and strengthening Somali institutions. In the education sector, USAID programmes have supported access to quality education, particularly for girls and marginalised communities, investing in teacher training and curriculum development through initiatives like the Bar Ama Baro programme, which enrolled over 100 000 students in its first year.^11 12^ A cessation in this support could undermine efforts to improve literacy rates and build a skilled workforce, essential for Somalia’s future economic development. Governance and stability initiatives, crucial for consolidating peace and fostering accountable institutions, have also been supported by USAID, including programmes aimed at strengthening local governance and promoting rules of law through projects like the US$40 million people-centred governance activities launched in 2023.^13^ Weakening these initiatives could jeopardise fragile political gains and potentially create vacuums that could be exploited by destabilising forces.^13 14^

Economic development programmes, designed to create livelihood opportunities and diversify Somalia’s economy beyond traditional sectors, have also benefited from USAID investment focusing on areas like agriculture and small enterprise growth.^15 16^ A decline in this support could hinder economic diversification efforts, limit job creation and perpetuate cycles of poverty and dependence on external aid. Crucially, sustained USAID engagement, despite its imperfections, has contributed to building local capacity within Somali institutions and civil society organisations. Withdrawal risks eroding this capacity and hindering the development of sustainable, Somali-led systems across various sectors. The overarching impact could be a significant setback to Somalia’s journey towards self-sufficiency and sustainable development, potentially reversing years of progress and entrenching dependence on external actors.

However, within this challenging scenario lies an undeniable opportunity, a forced but necessary catalyst for Somalia to fundamentally reimagine its development paradigm and prioritise greater self-reliance. While the immediate shock of reduced aid will be painful, it compels a critical examination of the long-term sustainability of aid dependence. For too long, Somalia’s development trajectory has been shaped by external funding and agendas, potentially undermining local ownership and fostering a culture of reliance rather than self-determination.^17 18^ This stage demands a strategic shift towards building endogenous capacity and mobilising domestic resources.

The path to self-reliance is multifaceted and requires rigorous action across various fronts: 1) Strengthening domestic revenue generation is paramount. This necessitates comprehensive tax reforms (eg, broadening the tax base beyond import duties, improving compliance, potentially digitising collection), improvements in tax collection efficiency, and enhanced governance and transparency to build public trust and attract both domestic and foreign investment. 2) Diversifying Somalia’s economy beyond traditional sectors like livestock and remittances is equally critical. Investing in sectors like renewable energy, technology, fisheries, and sustainable agriculture can create new livelihood opportunities and reduce vulnerability to external economic shocks. 3) Investing in human capital through education, skills training, and entrepreneurship is essential for building a productive and self-sufficient workforce. Prioritising quality education and vocational training aligned with the needs of a diversifying economy is crucial. 4) Good governance and accountability are the bedrock of sustainable development. Combating corruption, strengthening institutions and promoting transparency are essential for creating an enabling environment for economic growth, attracting investment and ensuring efficient resource allocation. 5) Engaging the Somali diaspora, a significant source of remittances and expertise, strategically is another vital avenue. This could involve creating secure investment approaches like diaspora bonds for infrastructure or establishing formal channels for skills transfer in critical sectors. 6) Exploring South-South cooperation with other developing nations that have successfully transitioned to greater self-reliance can provide valuable lessons and partnerships. 7) Empowering local civil society organisations and NGOs to play a leading role in development, fostering local ownership and accountability, is crucial for ensuring that development initiatives are truly responsive to Somali needs and priorities.

In navigating this challenging transition, both the Somali government and the international community have crucial roles to play. The Somali government must seize this moment to implement bold policy reforms aimed at strengthening domestic revenue, diversifying the economy and improving governance. This requires strong political will, strategic planning and inclusive dialogue with all stakeholders. For international partners, including those beyond USAID, the approach must shift from direct service delivery to supporting Somali-led initiatives, focusing on capacity building, technical assistance and flexible funding mechanisms that empower Somali institutions. This necessitates a move away from perpetuating dependence towards fostering partnerships that prioritise Somali ownership and sustainability. The global health community has a vital role in monitoring the health and humanitarian consequences of this policy shift, advocating for continued support to the most vulnerable and sharing evidence-based strategies for building resilient and self-reliant health systems in fragile states.

Successfully navigating this transition will require bold leadership, strategic reforms and a fundamental shift in both Somali and international approaches to development. The future of Somalia hinges on transforming this crisis into an opportunity to build a more resilient, self-sufficient and ultimately, independent nation, capable of charting its own course towards sustainable peace and prosperity. The journey will be complex and fraught with challenges, but the imperative for Somali ownership and self-determination has never been more urgent.

## Data Availability

There are no data in this work.

## References

[1] (2025). Freeze on foreign aid, usaid dismantling, and policy shifts threaten health programs worldwide. https://bayareaglobalhealth.org/alliance-news/freeze-on-foreign-aid-usaid-dismantling-and-policy-shifts-threaten-health-programs-worldwide/.

[2] OCHA (2024). Somalia 2024 humanitarian needs and response plan (HNRP). https://www.unocha.org/publications/report/somalia/somalia-2024-humanitarian-needs-and-response-plan-hnrp.

[3] ReliefWeb (2024). Somalia situation report, 13 aug 2024 - Somalia. https://reliefweb.int/report/somalia/somalia-situation-report-13-aug-2024.

[4] FA (2025). US foreign assisstance by country. https://foreignassistance.gov/.

[5] USAID Somalia (2015). Somali joint health and nutrition program. https://2017-2020.usaid.gov/news-information/fact-sheets/somali-joint-health-and-nutrition-program.

[6] UNICEF (2025). USAID provides $20 million to combat covid-19 and strengthen emergency services. https://www.unicef.org/somalia/press-releases/usaid-provides-20-million-combat-covid-19-and-strengthen-emergency-services.

[7] WHO EMRO (2025). USAID’s bha supports who to continue the health emergency response to Somalia’s drought. http://www.emro.who.int/ar/somalia/news/usaids-bha-supports-who-to-continue-the-health-emergency-response-to-somalias-drought.html.

[8] USUN Rome Mission (2022). USAID announces additional $411m to avert famine in Somalia. https://usunrome.usmission.gov/usaid-announces-additional-411m-to-avert-famine-in-somalia/.

[9] ReliefWeb (2019). USAID announces nearly $257 million in new humanitarian assistance for the people of Somalia - Somalia | reliefweb. https://reliefweb.int/report/somalia/usaid-announces-nearly-257-million-new-humanitarian-assistance-people-somalia.

[10] (2025). World vision and usaid launch transformative resilience program in Southwest Somalia to reduce aid dependency. https://www.wvi.org/stories/world-vision-and-usaid-launch-transformative-resilience-program-southwest-somalia-reduce.

[11] (2025). Rapid learning for lasting change: accelerated basic education in post-conflict, Somalia. https://inee.org/resources/rapid-learning-lasting-change-accelerated-basic-education-post-conflict-somalia.

[12] Cade W (2025). THE ministry of education’s bar ama baro program launches second year of classes on international literacy day after enrolling more than 100,000 students. https://moe.gov.so/en/the-ministry-of-educations-bar-ama-baro-program-launches-second-year-of-classes-on-international-literacy-day-after-enrolling-more-than-100000-students-2/.

[13] Somalia USE (2023). U.S. Launches new $40 million people-centered governance activity. https://so.usembassy.gov/usaids-new-40-million-people-centered-governance-activity/.

[14] Aidan Schneider (2025). Somalia: supporting good governance and community cohesion. https://dt-global.com/projects/tis-plus/.

[15] USAID Somalia (2020). Economic growth and trade | somalia | archive - U.S. agency for international development. https://2017-2020.usaid.gov/somalia/economic-growth-and-trade.

[16] DTGlobal (2025). Somalia: accelerating economic development for marginalized groups. https://dt-global.com/projects/iris/.

[17] (2025). Country brief on the humanitarian-development- peace nexus (Somalia). https://interagencystandingcommittee.org/sites/default/files/migrated/2021-09/Country%20Brief%20on%20the%20Humanitarian-Development-%20Peace%20Nexus%20%28Somalia%29.pdf.

[18] The New Humanitarian (2024). Somalia is changing. The humanitarian system must as well. https://www.thenewhumanitarian.org/opinion/2024/10/23/somalia-changing-humanitarian-system-must-well.

